# Retraction Note to: IL-6 signaling promotes DNA repair and prevents apoptosis in CD133+ stem-like cells of lung cancer after radiation

**DOI:** 10.1186/s13014-022-02030-5

**Published:** 2022-05-16

**Authors:** Yuhchyau Chen, Fuquan Zhang, Ying Tsai, Xiadong Yang, Li Yang, Shanzhou Duan, Xin Wang, Peter Keng, Soo Ok Lee

**Affiliations:** Department of Radiation Oncology, James P. Wilmot Cancer Center, University of Rochester, 601 Elmwood Ave., Box 647, Rochester, NY 14642 USA

## Retraction to: Radiation Oncology (2015) 10:227 10.1186/s13014-015-0534-1

The Editor-in-Chief has retracted this article following an investigation by the University of Rochester. The institution recommended retraction based on the following findings:

- Three of the immuno-fluorescent images in Figure 1C were wrong, and there are some remaining concerns about the quantification of the original data submitted for correction of that figure;
- There is an error in the original publication for a sphere image in Figure 2C;
- There is an error in the original publication for a cell culture image in Figure 3D.

Owing to the number and type of errors detected, as well as continuing concerns related to the interpretation of the data, the Editor-in-Chief no longer has confidence in the reliability of the work presented.

Yuhchyau Chen, Ying Tsai, and Peter Keng agree to this retraction. Fuquan Zhang, Xiadong Yang, Li Yang, Shanzhou Duan, Xin Wang and Soo Ok Lee have not responded to any correspondence from the editor or publisher about this retraction.

